# GC-TOF-MS-Based Metabolomic Analysis and Evaluation of the Effects of HX106, a Nutraceutical, on ADHD-Like Symptoms in Prenatal Alcohol Exposed Mice

**DOI:** 10.3390/nu12103027

**Published:** 2020-10-02

**Authors:** In Gyoung Ju, Mee Youn Lee, Seung Ho Jeon, Eugene Huh, Jin Hee Kim, Jong Kil Lee, Choong Hwan Lee, Myung Sook Oh

**Affiliations:** 1 Department of Life and Nanopharmaceutical Sciences, Graduate School, Kyung Hee University, Seoul 02447, Korea; igju801@khu.ac.kr (I.G.J.); eugenehuh@khu.ac.kr (E.H.); jinhee1231@khu.ac.kr (J.H.K.); 2 Department of Bioscience and Biotechnology, Konkuk University, Seoul 05029, Korea; kkamlice@daum.net; 3 Department of Fundamental Pharmaceutical Science, Graduate School, Kyung Hee University, Seoul 02447, Korea; bawoojang@khu.ac.kr; 4 Department of Medical Science of Meridian, Graduate School, College of Korean Medicine, Kyung Hee University, Seoul 02447, Korea; 5 Department of Pharmacy, College of Pharmacy, Kyung Hee University, Seoul 02447, Korea; 6 Department of Oriental Pharmaceutical Science, College of Pharmacy, Kyung Hee University, Seoul 02447, Korea

**Keywords:** attention deficit hyperactivity disorder, hyperactivity, nutraceutical, HX106, metabolomics

## Abstract

Attention deficit hyperactivity disorder (ADHD) is a neurodevelopmental disorder that occurs in children characterized by inattention and hyperactivity. Prenatal alcohol exposure (PAE) can disrupt fetal neuronal development and cause an ADHD-like hyperactive behavior in the offspring. In this study, we hypothesized that metabolic disturbance would involve in ADHD neuropathology and aimed to investigate the changes in metabolite profile in PAE-induced ADHD-like model and the effects of HX106, a nutraceutical, on ADHD-like pathophysiology and metabolite changes. To this end, we administered HX106 to the mouse offspring affected by PAE (OPAE) and assessed the hyperactivity using the open field test. We observed that HX106-treated OPAE showed less hyperactive behavior than vehicle-treated OPAE. The effects of HX106 were found to be related to the regulation of dopamine transporter and D2 dopamine receptor expression. Furthermore, using gas chromatography time-of-flight mass spectrometry-based metabolomics, we explored the metabolite changes among the experimental groups. The metabolite profile, particularly related with the amino acids, linoleic acid and amino sugar pathways, was altered by PAE and reversed by HX106 treatment partially similar to that observed in the control group. Overall, this study suggest that metabolite alteration would be involved in ADHD pathology and that HX106 can be an efficient supplement to overcome ADHD by regulating dopamine signaling-related protein expression and metabolite changes.

## 1. Introduction

Attention deficit hyperactivity disorder (ADHD) is a common neurodevelopmental disorder accompanied by inattention, impulse and hyperactivity that occurs in childhood and has a current prevalence of 5% in 4–17-year-old children [1,2]. It is known that prenatal alcohol exposure (PAE) leads to impaired neurodevelopment and dysfunction of the central nervous system in human and animal offspring [3,4,5]. PAE causes fetal alcohol spectrum disorders, which often co-occurs with ADHD at a ratio of over 60% and is considered as one of the etiological factors of ADHD [6]. Clinical, neuroimaging and genetic studies suggest that catecholamines, especially dopamine, play an important role in the development of ADHD [7,8]. Dopamine transmission occurs in the prefrontal cortex, nucleus accumbens and striatum, mediating attention, cognition, motor control and motivation with regard to the pathology of ADHD [9]. It is known that genetic variants of dopamine transmission-related factors, such as dopamine transporters and dopamine receptors contribute to the development of ADHD [10]. To control the symptoms of ADHD, psychostimulants regulating dopamine signaling are frequently prescribed including methylphenidate, which blocks dopamine transporters (DAT) and norepinephrine transporters and amphetamine, which indirectly activates dopamine receptor D1 and adrenoceptor α2 [11,12,13].

Metabolomics has been widely accepted as a useful tool to investigate altered metabolic pathways by analyzing the changes in small molecule metabolites (<650 Da) [14]. It can be applied to discover biomarkers, assess toxicity and explore efficacy and mechanisms of drugs containing multi-biochemical components [15]. This method provides an overview of the metabolic and biochemical pathways that enable to establish the basis of the pathophysiological and pharmacological understanding in multi-factorial neurological disorders, such as ADHD [14,15]. A number of studies have been conducted on several metabolic alterations, including amino acid and catecholamine metabolisms, in the serum or urine of ADHD patients [16,17,18,19,20,21,22,23]. However, metabolite changes in PAE-induced ADHD-like symptoms are obscure.

HX106 is a nutraceutical composed of four different herbal extracts: Arilli of *Dimocarpus longan* Loureiro (Sapindaceae), tubers of *Liriope platyphylla* Wang and Tang (Liliaceae), roots of *Salvia miltiorrhiza* Bunge (Lamiaceae) and rhizomes of *Gastrodia elata* Blume (Orchidaceae). These compositions were selected and mixed according to the traditional knowledge and information from previous studies [24,25]. HX106 has been reported to improve memory function possibly mediated by the suppression of oxidative stress, neuroinflammation and accumulation of amyloid beta and tau tangle [24,25,26]. Based on these results, HX106 has been applied into clinical study (NCT01956097). These data suggest that HX106 can be an effective regulator for neurological disorders. In this study, we aimed to assess the effects of HX106 on PAE-induced ADHD-like phenotype and metabolic changes in the offspring mice. First, we examined the therapeutic efficacy of HX106 in the offspring affected by PAE (OPAE), focusing on hyperactivity. Then, we investigated the changes in protein expression of dopamine signaling-related proteins including DAT and D2 dopamine receptor (DRD2) in the brain of the offspring. In addition, we applied metabolomics to analyze the metabolic perturbation of ADHD and the regulation after HX106 treatment. We performed untargeted analysis on the alterations in metabolites by applying gas chromatography time-of-flight mass spectrometry (GC-TOF-MS) in the plasma, liver, colon and feces of the offspring of PAE mouse.

## 2. Materials and Methods 

### 2.1. Materials

HX106 were prepared as previously described [24] and provided by Helixmith Co., Ltd. (Seoul, Korea). Radioimmunoprecipitation assay buffer (RIPA) buffer (89901) and protease/phosphatase inhibitor cocktail (78445) were obtained from Thermo Fisher Scientific (Waltham, MA, USA). Mouse anti-β-actin (sc-47778 HRP) and horseradish peroxidase (HRP)-conjugated secondary antibodies against rat or rabbit IgG were purchased from Santa Cruz Biotechnology (Dallas, TX, USA). Rabbit anti-DRD2 was purchased from Cell Signaling Technology (Danvers, MA, USA). Rabbit anti-rat IgG (biotinylated), avidin-biotin complex (ABC) mixture and normal rabbit serum were purchased from Vector Laboratories (Burlingame, CA, USA). Rabbit anti-DAT (AB1591P) and polyvinylidene difluoride (PVDF) membranes were purchased from Millipore (Burlington, MA, USA). Tetramethylethylenediamine, protein assay reagent, acrylamide and enhanced chemiluminescence (ECL) reagent were purchased from Bio-Rad Laboratories (Hercules, CA, USA). Triton X-100, paraformaldehyde (PFA), phosphate buffer (PB), phosphate-buffered saline (PBS, pH 7.4), tris-buffered saline (TBS), 3,3’-diaminobenzidine (DAB), DPX mounting medium and the other reagents unnoted were purchased from Sigma-Aldrich (St. Louis, MO, USA).

### 2.2. Animals and Treatment

The female ICR mice (28–30 g weight) was purchased from Daehan Biolink (Eumseong, Chungcheongbuk-do, Republic of Korea) at gestation day (GD) 3 and housed in plastic cages under constant temperature (23 ± 1 °C) and humidity (50 ± 10%), in a 12 h light/dark cycle with free access to food and water. Stabilized animals were divided into three groups and two groups were treated with ethanol half-diluted with autoclaved saline (EtOH; 6 g/kg/day; 50 *v*/*v*%) from GD 6 to GD 15 via oral gavage. Control groups were treated with saline (0.9% NaCl). The treatment was divided into two equal doses 10 h intervals at 10:00 and 20:00. The day after birth was considered postnatal day (PD) 1. The offspring was separated at PD 21 (*n* = 10 per each group). Saline was administered to the control and OPAE groups and HX106 dissolved in saline was treated at 200 mg/kg/day to the HX106-treated OPAE group by oral gavage. The administration was proceeded for 3 weeks. All animal studies were performed in accordance with the “Guide for the Care and Use of Laboratory Animals, 8th edition” (National Institutes of Health, 2011) and approved by the Animal Care and Use Guidelines Committee of Kyung Hee University (KHUASP(SE)-19-013).

### 2.3. Open Field Locomotor Tests

The open field locomotor test was performed between 8 p.m. and 10 p.m. to avoid variation. The mice were placed in the testing chambers (40 × 25 × 18 cm) with black floors, followed by a 20-min recording period using a computerized automatic analysis system (Viewer; Biobserve, Bonn, Germany). The data collected using the computer included the total distance traveled, by tracking the center of the animal. During the behavioral experiment, the movement of the mouse detected by the program was continuously monitored by the experimenters.

### 2.4. Preparation of Animal Tissues

Mice were euthanized after behavioral testing by administration of a mixture of ketamine and xylazine in saline as the anesthetic. Then, blood was collected from the heart and placed in EDTA-treat blood tube to prevent clotting. After centrifugation at 3000 rpm for 5 min, the supernatant (plasma) was collected and used. Both colon and liver were obtained from anesthetized mice and feces were taken from the colon. The tissues were immediately placed in a tube and stored in deep freezer (−70 °C). For immunohistochemistry, half of the mice in each group were perfused transcardially using 4% PFA in 0.1 M PB. After perfusion, brains were removed, post-fixed by PFA overnight at 4 °C and incubated in 30% sucrose at 4 °C until dehydration. Sequential 25-μm-thick coronal sections were prepared by a cryostat (CM1850; Leica, Wetzlar, Germany) and stored at −20 °C. For western blotting, the striatum and cortex were obtained from the brains of the last half in each group through dissection.

### 2.5. Immunohistochemistry

Free-floating brain sections were washed in PBS before immunostaining and then treated with 1% hydrogen peroxide for 15 min to get rid of endogenous peroxidase activity. Then, the sections were incubated with a rat anti-DAT (1:1000 dilution) in PBS containing 0.3% Triton X-100 and normal rabbit serum overnight. After the brain sections were incubated with a biotinylated anti-rat IgG (1:200 dilution) for 1 h, the sections were gently incubated with ABC solution for another 1 h at room temperature. The color was developed with DAB for 5 min. The tissue sections were mounted on gelatin-coated slides, air dried, dehydrated with ascending alcohol concentrations, cleared with xylene and cover slipped using DPX histomount medium.

### 2.6. Western Blot Analysis

Western blot analysis was performed as previously described [27]. The brain tissues were homogenized in RIPA buffer with protease/phosphatase inhibitors. Equal amounts of protein samples (20 μg) in sodium dodecyl sulfate (SDS) sample buffer were separated by SDS polyacrylamide gel electrophoresis and transferred to PVDF membranes by electrophoresis. The membranes were pretreated with blocking solution (5% bovine serum albumin plus 0.1% tween 20 in TBS) for 1 h at room temperature and incubated with primary antibodies: rat anti-DAT, anti-DRD2 (1:1000), respectively in blocking solution overnight at 4 °C. The membranes were washed five times with washing buffer (0.1% tween 20 in TBS) for 10 min each and incubated with HRP-conjugated secondary antibodies against rat or rabbit IgG in blocking solution for 1 h. The membranes were washed five times for 10 min each with washing solution and protein detection was carried out using an ECL reagent and visualized by ChemiDoc (Vilber Lourmat, Paris, France). The intensity of bands was quantified using Image J software (National Institutes of Health, Bethesda, MD, USA). For the quantification of relative protein expression, the optical density of the protein band of interest was normalized to the optical density of β-actin detected on the same membrane. 

### 2.7. Extraction of Animal Sample for Metabolomics

The plasma samples were extracted by adding 1 mL of ice-cold 100% methanol to 200 μL of mouse plasma. Frozen liver (130 mg), colon (~100 mg) and feces (~100 mg) were added 100% methanol. Each mixture samples were homogenized (frequency = 30 Hz) for 10 min by using a Retsch MM400 mixer mill (Retsch GmbH & Co, Haan, Germany). Then, the sample was centrifuged at 4°C and 13,000 rpm for 10 min. The supernatants were further passed through a 0.2-μm polytetrafluoroethylene syringe filter and finally transferred to Eppendorf tubes. The supernatant was completely dried with a speed vacuum machine and stored in a −80 °C. Dried extracts were reconstituted with 100% methanol to a final concentration of 10 mg/mL for instrument analysis. The samples were again dried using a speed vacuum concentrator prior to a two-staged derivatization step.

For the GC-MS analysis, the re-dried sample was oximated with 50 μL of methoxyamine hydrochloride in pyridine (20 mg/mL) for 90 min at 30 °C using a thermomixer (Eppendorf, Hamburg, Germany). Then, the oximated samples were silylated with 50 μL of *N*-Methyl-*N*-(trimethylsilyl) trifluoroacetamide (MSTFA) for 30 min at 37 °C, using a thermomixer. The pooled quality control (QC) samples were prepared from 50 μL blends of each sample. The analytical samples were analyzed in blocks of seven runs followed by an intermittent QC analysis to ensure the data quality and method’s robustness.

### 2.8. GC-TOF-MS Analysis

The GC-TOF-MS analyses were performed using an Agilent 4890 GC system (Palo Alto, CA, USA) coupled with a Leco TOF Pegasus III mass spectrometry. Metabolites were separated by an DB-5MS column (30 m × 0.25 mm I.D. × 0.25 µm, J & W Scientific, Folsom, CA, USA) was used with helium as the carrier gas at a constant flow rate of 1.5 mL/min. A total of 1 μL of the derivatized sample was injected in split less mode. The oven temperature was maintained at 75 °C for 2 min, then increased to 300 °C at a rate of 15 °C/min and held for 3 min. The mass data were collected in the electron ionization (EI) mode with ionization energy of 70 eV and mass scan (*m/z*) range of 50–1000 at an acquisition rate of 20 spectra/s. The injector and ion source temperatures were set at 250 and 230 °C, respectively.

### 2.9. Data Processing and Statistical Analyses

Behavioral and histological analysis data were expressed as the mean ± standard error of the mean (SEM) using GraphPad Prism 8.0 software (GraphPad software Inc., San Diego, CA, USA). The results were analyzed statistically by one-way analysis of variance (ANOVA) or two-way ANOVA followed by Tukey’s multiple comparisons test. A value of *p* < 0.05 was considered statistically significant. The GC-TOF-MS raw data processing and multivariate statistical analysis were conducted as described in our previous study [28]. Raw data were converted to a NetCDF format (*.cdf) using ChromaTOF (version 4.44, LECO). After conversion, the MS data were processed using the Metalign software package (http://www.metalign.nl) to obtain a data matrix containing retention times, accurate masses and normalized peak intensities. The resulting data were exported to Excel (Microsoft, Redmond, WA, USA) for multivariate data analysis. Multivariate data analyses were performed using the SIMCA-P+ software (version 12.0, Umetrics, Umea, Sweden). Principal component analysis (PCA) and partial least squares discrimination analysis (PLS-DA) were performed to compare the different experimental groups. The significantly discriminant metabolite with a variable importance in projection (VIP) value exceeding 1.0 was obtained using the PLS-DA model. Significant differences were tested by one-way ANOVA using the STATISCA program (version 7.0, StaSoft Inc., Tulsa, OK, USA). As a post-hoc test, Tukey’s honestly significant difference test was employed. The metabolites detected by GC-TOF-MS were putatively identified using standard compounds, an in-house library and databases of the National Institute of Standards and Technology (NIST MS Search Program, version 2.0, Gaithersburg, MD, USA) by comparing their retention time and mass spectrometry data. We imported the datasets of differential metabolites from plasma, liver, colon and feces to an open source, web-based software, Metaboanalyst 4.0 (McGill University, Montreal, QC, Canada; http://www.metaboanalyst.ca) to draw the heatmaps and apply the pathway analysis module. The data sets of peak height intensity were filtered by interquartile range and normalized by quantile. For the pathway analysis, Kyoto Encyclopedia of Genes and Genomes (KEGG) database were used as reference metabolic pathways.

## 3. Results

### 3.1. Effects of HX106 on Offspring Hyperactivity 

Pregnant mice were orally administered with EtOH from GD 6 to GD 15. To determine whether EtOH exposure during pregnancy caused serious developmental problems in the offspring, postnatal growth retardation was assessed after weaning (PD21). A significant weight loss at week 5 was observed in the OPAE group when compared to that in the control group. HX106 treatment started from week 3 gradually improved the weight loss; however, it was not significantly different from the OPAE group (Table 1). Meanwhile, the body weights of dams treated with and without EtOH during pregnancy did not differ from each other (Figure S1).

**Figure 1:**
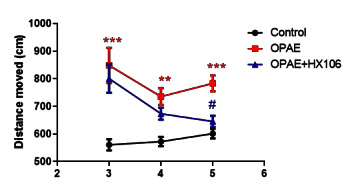
Effects of HX106 on hyperactivity induced by PAE. Total distance moved was measured by open field locomotor test. The data were analyzed by two-way ANOVA followed by Tukey’s multiple comparisons test. ** *p* < 0.01 and *** *p* < 0.001 vs. Con; # *p* < 0.05, vs. OPAE group. Con; the control group, OPAE; the offspring affected by PAE.

We investigated the hyperactivity of the offspring with or without PAE using the open field test at 3, 4 and 5 weeks from the birth. The distance moved was increased in the OPAE group than in the control group in all the weeks assessed. At week 3, the distance moved by the HX106-treated OPAE group was higher than that of the control group and did not differ from the OPAE group. After treatment, the distance moved by the HX106-treated OPAE group was significantly decreased compared to that of the OPAE group (Figure 1). We also performed the y-maze test to assess the ability on working memory and concentration and found no difference in behavior among all the groups (Figure S2).

### 3.2. Effects of HX106 on Dopamine Signaling-Related Factors in the Striatum of Offspring

**Figure 2:**
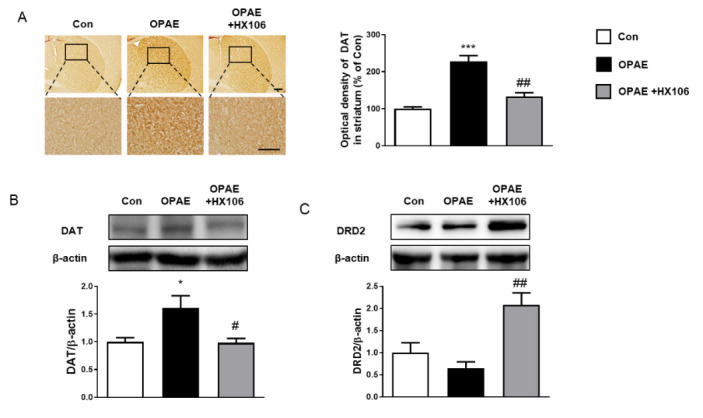
Effects of HX106 on expressions of dopamine transporter (DAT) and dopamine D2 receptor (DRD2) in the striatum of offspring at week 5. (**A**) DAT expression in the striatum was visualized using 3,3’-diaminobenzidine staining. The representative photographs and the quantifications are shown. Scale bar = 50 μm (**B**) The protein expression of DAT in the striatum. (**C**) The protein expression of DRD2 in the striatum. The data were analyzed by one-way ANOVA followed by Tukey’s multiple comparisons test. * *p* < 0.05 and *** *p* < 0.001 vs. Con; # *p* < 0.05 and ## *p* < 0.01 vs. OPAE group. Con; the control group, OPAE; the offspring affected by PAE.

We next examined the effects of HX106 on the protein expression of DAT in the striatum of the OPAE group. By performing immunohistochemistry, we observed that the level of DAT increased in the OPAE group, whereas it decreased in the HX106-treated group (Figure 2A). Following western blot analysis, we confirmed the results that the expression of DAT was shifted in the OPAE group and reversed in the HX106-treated group in the striatum (Figure 2B). In addition, we measured DRD2 expression levels in the striatum. No difference was observed in the levels of DRD2 between control and OPAE groups; however, we found that DRD2 level was significantly elevated in the HX106-treated group (Figure 2C).

### 3.3. Effects of HX106 on Metabolite Profiles Altered by Pae in Mouse Plasma, Liver, Colon and Feces

**Figure 3:**
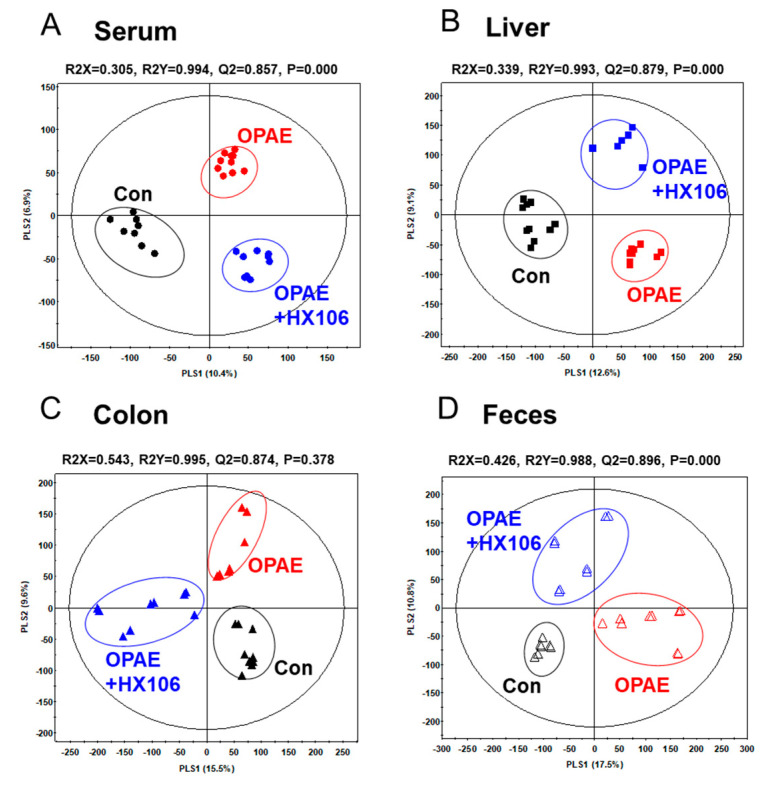
Partial least-squares discriminant analysis (PLS-DA) score plot based on metabolic profiling of plasma (**A**), liver (**B**), colon (**C**) and feces (**D**). Con; the control group, OPAE; the offspring affected by PAE.

In order to investigate whether endogenous metabolites were altered by the HX106 treatment, we performed the metabolite profiling in the plasma, liver, colon and feces of the offspring at week 5 using GC-TOF-MS. Multivariate analyses including the unsupervised PCA (Figure S3), as well as the supervised PLS-DA were performed to select the discriminant metabolites adding to the observed variance. The PLS-DA score plots revealed that all the three groups were distinct from each other with statistically significant model values viz., R2X(cum), R2Y(cum) and Q2 (cum), indicating its fitness and prediction accuracy at a *p*-value obtained through cross-validation (Figure 3). In the PLS-DA score plot of plasma, liver, colon and feces showed clear division of the three groups (control, OPAE, HX106-treated OPAE) which are discriminated by PLS component 1 and 2. The results indicate that PAE and HX106 administration caused significant differences in endogenous metabolite levels.

### 3.4. Identification of the Discriminant Metabolites

To investigate the effect of HX106 administration on prenatal alcohol-exposed mice, the first principal component of the VIP was obtained by assessing the influence of every term in the matrix variable X on all the variable Y’s, where X and Y indicate the time and metabolite, respectively. VIP was normalized so that Sum (VIP)2 = K (number of terms in the matrix X.) VIP values >1.0 were selected as changed metabolites initially. Based on the aforementioned analysis, 29 metabolites in plasma (Table S1), 31 metabolites in liver (Table S2), 32 metabolites in colon (Table S3) and 34 metabolites in feces (Table S4) were listed as significantly altered.

**Figure 4:**
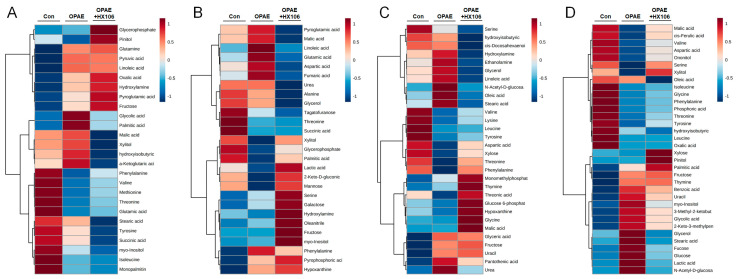
The hierarchical clustering heat map visualizing the altered metabolites in plasma (**A**), liver (**B**), colon (**C**) and feces (**D**). Con; the control group, OPAE; the offspring affected by PAE.

To visualize the alteration in metabolites among the three groups, we constructed the hierarchical clustering heat map in accordance with the relative quantities of the altered metabolites with VIP value >1.0. As shown in Figure 4, altered metabolites caused by OPAE and HX106 treatment were indicated by color differences. The metabolites in the OPAE group including amino acids, organic acids and fatty acids, were markedly different from that in the control group, whereas a significant portion of them was altered after HX106 treatment.

### 3.5. Pathway Analysis Associated with the Altered Metabolites

**Figure 5:**
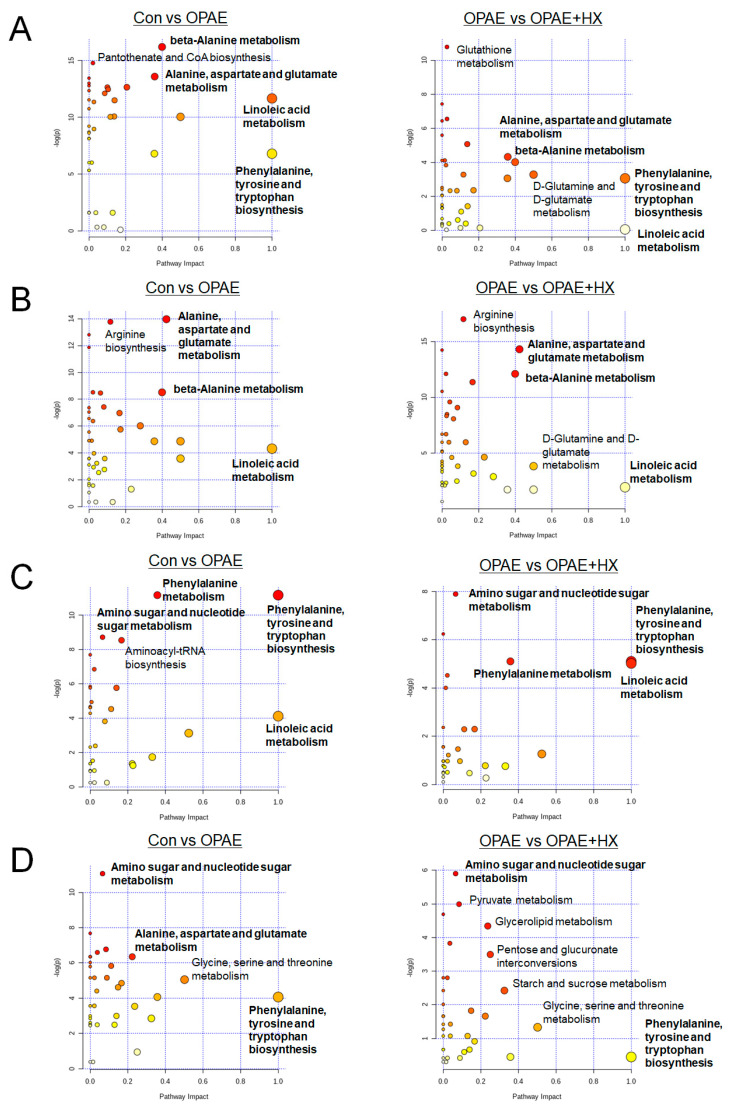
Pathway analysis of the altered metabolites in plasma (**A**), liver (**B**), colon (**C**) and feces (**D**) visualized by bubble plots. OPAE; the offspring affected by PAE, OPAE + HX; HX106-treated OPAE group.

**Figure 6:**
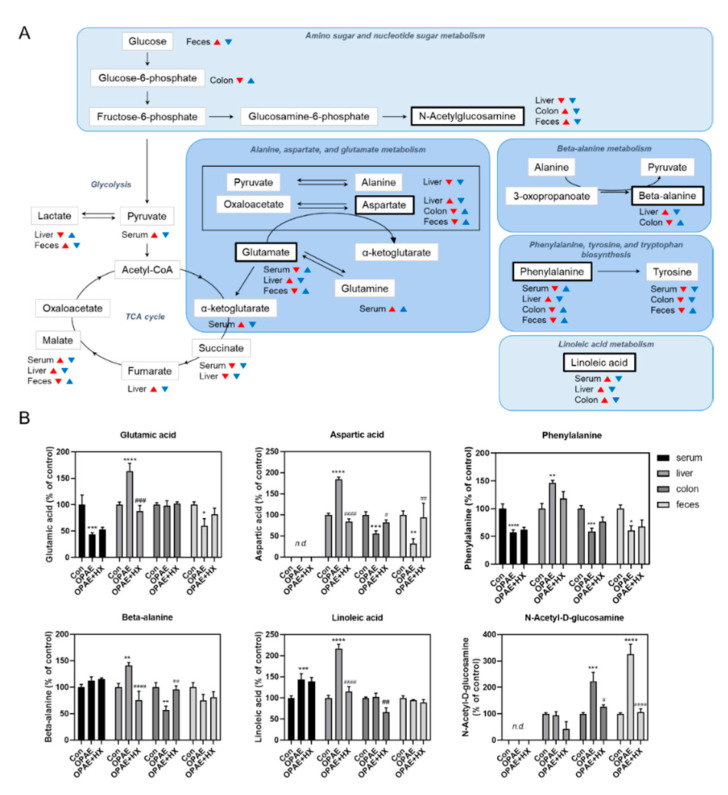
The metabolic pathways and the changes of main metabolites in plasma, liver, colon and feces. Red arrow presents the alterations in the OPAE group compared to the control group while blue arrow presents the alterations in HX106-treated OPAE group compared to the OPAE group (VIP value > 1.0) (**A**). The relative levels of key metabolites are shown (**B**). The data were analyzed by one-way ANOVA followed by Tukey’s multiple comparisons test. * *p* < 0.05, ** *p* < 0.01, *** *p* < 0.001 and **** *p* < 0.0001 vs. Con; # *p* < 0.05, ## *p* < 0.01, ### *p* < 0.001 and #### *p* < 0.0001 vs. OPAE group. Con; the control group, OPAE; the offspring affected by PAE, OPAE + HX; HX106-treated OPAE group, n.d.; not detected.

We performed pathway analysis with the altered metabolites that are mentioned above using the MetaboAnalyst 4.0 software. The important pathways affected by the PAE and HX106 treatment were identified and are shown in Figure 5. The pathway alterations were appeared similarly between plasma and liver or between colon and feces. In specific, in the plasma and liver samples, alanine, aspartate and glutamate metabolism and beta-alanine metabolism were noted to be significantly altered by both the PAE and HX106 treatment. On the other hand, phenylalanine, tyrosine and tryptophan biosynthesis and linoleic acid metabolism were highlighted in the colon sample, while amino sugar and nucleotide sugar metabolism were emphasized in both the colon and fecal samples. Using bold fonts, we highlighted the pathways altered simultaneously in two or more tissues. The map of mainly changed metabolism pathways and the alterations of key metabolites are shown in Figure 6. The amino acid pathways were shown in the bluer color.

## 4. Discussion

To date, ample evidence has shown that parental alcohol abuse during the perinatal period may induce ADHD-like phenotypes in offspring such as hyperactivity and inattention [29]. In this study, we aimed to find out metabolic alterations in PAE-induced ADHD-like model and to investigate the recovery of phenotypes after the HX106 treatment, a nutraceutical previously developed to improve cognitive function. We administered EtOH to pregnant mice from GD 6 to GD 15, which is the period for neural differentiation and confirmed that the hyperactive behavioral phenotype appeared in the offspring [30,31]. After administering the HX106 to the offspring, we observed that hyperactivity was alleviated in the HX106-treated offspring group, suggesting that HX106 can improve the ADHD-like behavior. Meanwhile, we conducted a y-maze to investigate the working memory function and spontaneous alternation behavior requiring attention as shown in the previous study [32]. However, unlike the reported results, there was no difference between the control and OPAE groups (Figure S2). Further studies would be needed to investigate and establish the phenotype changes reflecting inattention and memory impairment in animal models.

Although the exact etiology of ADHD is ambiguous, the view that factors related with dopamine signaling are associated with ADHD pathophysiology is widely accepted because the mechanisms of action of drugs currently used for ADHD patients, such as methylphenidate and amphetamine are known to increase synaptic dopamine transmission [12]. Since methylphenidate blocks DAT, many studies have been conducted to reveal the relationship between DAT and ADHD and to explore how to regulate DAT function as a therapeutic approach [33,34]. It has been reported that specific missense mutation of the human DAT coding gene is a risk factor of ADHD, suggesting that DAT is involved in ADHD pathology [35]. In the striatum of ADHD patients, the expression of DAT was revealed to be upregulated, implying that the removal of dopamine was increased in the synaptic cleft [13]. Meanwhile, Chen et al., suggested taurine as a regulator of ADHD symptoms, demonstrating that the ameliorating effects on ADHD-like behavior are associated with reduced DAT expression in a spontaneously hypertensive rat model [34]. Contrarily, it was reported that hypofunction of DAT results in ADHD-like behavior demonstrated by gene manipulation in mice [36]. These controversial results make it difficult for studies to draw a unified phenotype of DAT expression in the brains with ADHD-like behavioral phenotype. Nevertheless, in the mouse model representing ADHD-like behavior induced by PAE, Kim et al., proved that the expression of DAT was upregulated in striatum similar to the results reported in human ADHD [4]. In this study, we also measured DAT expression in the striatum using immunohistochemistry and western blot analyses and confirmed that the expression levels of DAT were upregulated in the OPAE group as expected. By observing that the expression levels of DAT in the HX106-treated group were significantly reduced compared to the OPAE group, we could determine that the effects of HX106 on hyperactivity are related to reduced DAT expression possibly followed by dopamine removal and the reduction of dopamine signaling. 

Several studies have investigated the role of DRD2 in pharmacological mechanisms to treat ADHD. Reportedly, activation of DRD2 stimulates a signaling cascade including Akt/glycogen synthase kinase 3 signaling, leading to decrease locomotor activity [37,38]. It has been proven that the ameliorative effect of amphetamine on hyperactivity is associated with an increase in dopamine amount in the synaptic cleft and the stimulation of dopamine signaling mediated by DRD2, by showing that DRD2-selective blockade reduced the effects of amphetamine [39]. In this regard, the activators of DRD2 are considered to be good candidates to regulate the hyperactive behavior. In this study, we measured DRD2 protein levels and observed that the expression was significantly increased in the HX106-treated OPAE group compared to the OPAE group. These data suggest that HX106 would activate DRD2-mediated dopamine signaling by increasing DRD2 expression, resulting in the attenuation of the hyperactive behavior. 

Understanding metabolite changes in the pathological status and after medication may contribute to elucidate the pathophysiology and to discover the drug targets and metabolic pathway regulated by the medications. Accumulated evidence has shown that there are significant alterations in metabolic profile proved by analyzing the biological samples from ADHD patients. The reported alterations in amino acid metabolism can be characterized by changes in tryptophan/kynurenine metabolism, which representatively showed an increase of 3-hydroxykynurenine concentrations in the plasma of ADHD patients [17,18,19,40]. On the other hand, melatonin metabolism, monoamine metabolism and glutamate metabolism pathways are also repeatedly investigated in the brain or urine of ADHD patients [20,23,41,42,43,44,45,46]. In this study, in order to investigate the metabolite changes involved in the neuropathology of ADHD-like hyperactivity induced by PAE and the ameliorative effects of HX106 treatment, we performed untargeted metabolomics using GC-TOF-MS. We clearly presented that the metabolite profiles in plasma, liver, colon and feces were altered in the OPAE group and that the changed direction was reversed by the HX106 treatment. These results suggest that the specifically increased or decreased metabolites may affect brain development, consequently leading to aggravation or attenuation of the ADHD symptoms. 

We observed alterations in various amino acid metabolism pathways in plasma, liver, colon and feces which was generally reduced in OPAE mice and increased by the HX106 treatment. By utilizing pathway analysis, we could determine that alanine, aspartate and glutamate metabolism, beta-alanine metabolism and phenylalanine metabolism are mainly regulated by the amino acid metabolism pathway after HX106 treatment. Particularly, phenylalanine, beta-alanine, aspartate and glutamate showed significant changes. Our data imply that the altered amino acids may be related to the hyperactive behavior induced by PAE and the ameliorating effects of HX106 and provide a novel point of view on the treatment of ADHD neuropathology. Reportedly, glutamatergic increases in the anterior cingulate cortex and positively correlates with hyperactivity and impulsivity [44]. Also, the ratio of glutamate to glutamine is known to decrease in the dorsolateral prefrontal cortex and basal ganglia of adult ADHD patients [45]. Beta-alanine is reported to possess attenuating effects on morphine-induced hyperactivity, which may be related to its property mimicking gamma-aminobutyric acid [47]. Phenylalanine is a well-known precursor of neurotransmitter, typically considered to play a role in neurological disorders. Our data showed that phenylalanine concentration decreased by PAE in plasma, colon and feces, whereas that increased in liver. Indeed, it is reported that children exposed to an environment with elevated levels of phenylalanine, such as phenylketonuria, are associated with ADHD symptoms [48]. On the other hand, phenylalanine concentration is reported to increase in dogs with ADHD-like symptoms [49]. These reports suggest that amino acids metabolism would involve in the neuropathology of ADHD-like hyperactivity; however, the report that phenylalanine increases in plasma is not consistent with our result. Hence, the role of phenylalanine in ADHD pathology should be studied in future studies.

Recently, there are some efforts to control neurological disorders by applying the gut-brain axis theory, a bidirectional communication between the brain and gastrointestinal tract [50,51]. In cases of ADHD, there is evidence demonstrating that gut microbiota affects brain neurodevelopment [52,53,54,55]. For instance, Szopinska-Tokov et al. suggested that inattentive behavior that appeared in ADHD patients is associated with a specific bacterium [53]. Also, it is reported that colonizing microbiota from ADHD patients to experimental mice affects the alteration of brain structures and causes concomitant changes in the behavior [55]. In this study, we investigated intestinal perturbation in a PAE-induced ADHD-like mouse model and the regulatory effects of HX106 on the gut microenvironment by analyzing metabolite changes in colon and fecal samples. We observed that there are noteworthy changes in metabolite profile, including amino sugar metabolism and amino acids metabolism. This is the first time to present the alteration in gut microenvironment caused by PAE that leads to the hyperactive behavior of the offspring, implying that gut metabolites may be implicated in the neuropathology of ADHD. Additionally, we analyzed the correlation between metabolite alteration and behavioral change and revealed that fecal metabolites showed a strong relationship with the hyperactive behavior (Figure S4). Although the analyzed sample size was small, it was clearly shown that several types of amino acids are negatively correlated with hyperactivity, whereas amino acid metabolites including branched chain α-keto acids are positively correlated. These data imply that fecal metabolites can reflect the pathological status of ADHD; however, additional further study would be warranted to elucidate the detailed role of gut microenvironment in ADHD pathophysiology. 

When analyzing pathways other than amino acid metabolism, we also observed that the levels of linoleic acids in that plasma, liver and colon was significantly changed by PAE or HX106 treatment. Our data supports the previous study which reported that linoleic acid increased in the blood samples of adolescents with an ADHD assessed in a case-control study [56]. On the other hand, in colon and fecal samples, we observed the alteration in amino sugar pathway highlighted by the change of N-acetyl-D-glucosamine. We report the increase of N-acetyl-D-glucosamine in ADHD-like model for the first time. To clarify the alteration of both fatty acid metabolism and amino sugar in ADHD, more studies should be accumulated. Although the role of linoleic acid and N-acetyl-D-glucosamine is not clear yet, we suggest that the alterations of linoleic acid and N-acetyl-D-glucosamine which remarkably increased in the OPAE group and was reversed by HX106 administration partially similar to the control group might support the normalizing effects of HX106 in ADHD pathophysiology. 

The efficacy of HX106 on ADHD-like symptoms was investigated in this study for the first time. Although HX106 has been originally developed to enhance cognitive function, our results speculate that the application of HX106 might be extended to ADHD patients for attenuation of the hyperactivity. When we searched the previous studies to figure out which of the four ingredients contributed to the effects of HX106, there was no study reporting an efficacy of each ingredient on ameliorating ADHD symptoms. However, among the four ingredients of HX106, rhizomes of *G. elata* are known to inhibit monoamine metabolism including dopamine, resulting in the attenuation of depressive disorder [57,58]. Also, roots of *S. miltiorrhiza* are reported to increase dopamine release in rat and PC12 cell lines and to mimic the action of amphetamine [59,60,61]. Considering these previous reports, we could assume that rhizomes of *G. elata* and roots of *S. miltiorrhiza* are the main players of HX106 on dopamine signaling-related regulation. 

Taken together, the present study provided a novel perspective on ADHD-like phenotypes in terms of metabolic changes and demonstrated the effects of HX106 on ADHD-like symptoms. The administration of HX106 clearly ameliorated the hyperactive behavior and regulated the expressions of DAT and DRD2. Moreover, by performing metabolomics analysis, we showed that PAE disrupted amino acid metabolism, linoleic acid metabolism and amino sugar metabolism and the altered metabolite profile was recovered by HX106 treatment. These results suggest that ADHD pathology may involve metabolic disruptions and that HX106 can be a promising supplement to attenuate the hyperactivity appears in PAE-induced ADHD-like neuropathology.

## Figures and Tables

**Table 1:** The body weight of the offspring with or without prenatal alcohol exposure (PAE). Changes of the body weight of offspring during HX106 treatment. The body weight of offspring was measured once a week at 3, 4 and 5 weeks from birth. The data were analyzed by two-way analysis of variance (ANOVA) followed by Tukey’s multiple comparisons test. * *p* < 0.05 and ** *p* < 0.01 vs. Con. Con; the control group, OPAE; the offspring affected by PAE.

Groups	Week 3	Week 4	Week 5
Con	12.05 ± 0.66	21.35 ± 0.54	25.70 ± 0.53
OPAE	10.55 ± 0.88	19.00 ± 0.92	22.33 ± 0.75 **
OPAE + HX106	9.05 ± 0.89 *	17.45 ± 0.88 **	23.16 ± 0.78

## Supplementary Materials

The following are available online at https://www.mdpi.com/2072-6643/12/10/3027/s1, Figure S1: The body weight of pregnant dams in gestation day 15, Figure S2: Effects of HX106 on memory and concentration, Table S1: List of discriminated metabolites identified in the plasma of prenatal alcohol-exposed mice treated with HX106, Table S2: List of discriminated metabolites identified in the liver of prenatal alcohol-exposed mice treated with HX106, Table S3: List of discriminated metabolites identified in the colon of prenatal alcohol-exposed mice treated with HX106, Table S4: List of discriminated metabolites identified in the feces of prenatal alcohol-exposed mice treated with HX106, Figure S3: Principal component analysis (PCA) score plot based on metabolic profiling of plasma, liver, colon and feces, Figure S4: Correlation plot between fecal metabolites with hyperactive behavior.
