# Supplementary material for: GC-TOF-MS-Based Metabolomic Analysis and Evaluation of the Effects of HX106, a Nutraceutical, on ADHD-Like Symptoms in Prenatal Alcohol Exposed Mice
Source: Nutrients. 2020 Oct 2;12(10):3027. doi: 10.3390/nu12103027 (PMC7600704; doi:10.3390/nu12103027)
Supplement: Supplementary file 1 [file nutrients-12-03027-s001.pdf]

nutrients

Supplementary methods

Y-maze

The Y-maze is three-arm-horizontal maze of which arms are disposed at 120° angles from each other (40 cm long and 3 cm wide with 12 cm high walls). The constituent of the maze floor and walls has been described previously [1]. Each arm has the sequence like A, B and C and mice were placed within any arm. Numbers of arm entries were recorded manually for each mouse over an 8 min. period. An actual alternation was defined as entries into all three arms on consecutive choices like ABC, CAB, or BCA. Maze arms were thoroughly cleaned between tasks to remove residual odors. The result was expressed as percentage of alternation to the following equation: % Alternation = [(Number of alternations) / (Total arm entries-2)] × 100.

Supplementary results

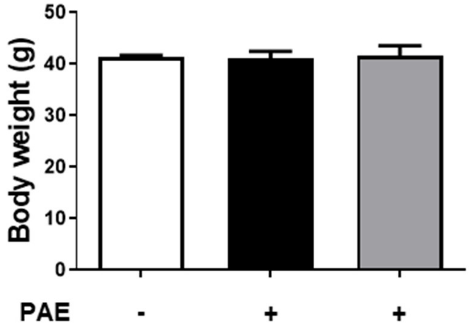

Figure S1. The body weight of pregnant dams in gestation day (GD) 15. The data were analyzed by one-way analysis of variance (ANOVA) followed by Tukey's multiple comparisons test. PAE; prenatal alcohol exposure.

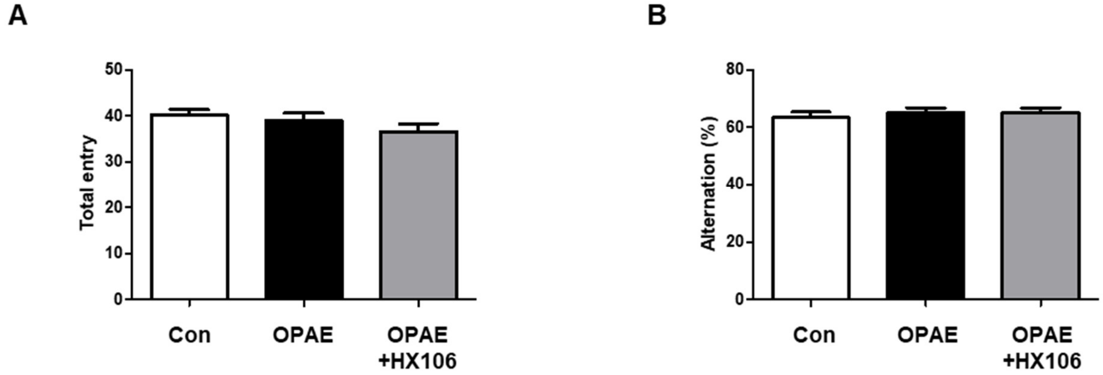

Figure S2. Effects of HX106 on memory and concentration. (A) total entry and (B) spontaneous alternation were measured by Y-maze test. The data were analyzed by one-way ANOVA followed by Tukey's multiple comparisons test. Con; the control group, OPAE; the offspring affected by PAE.

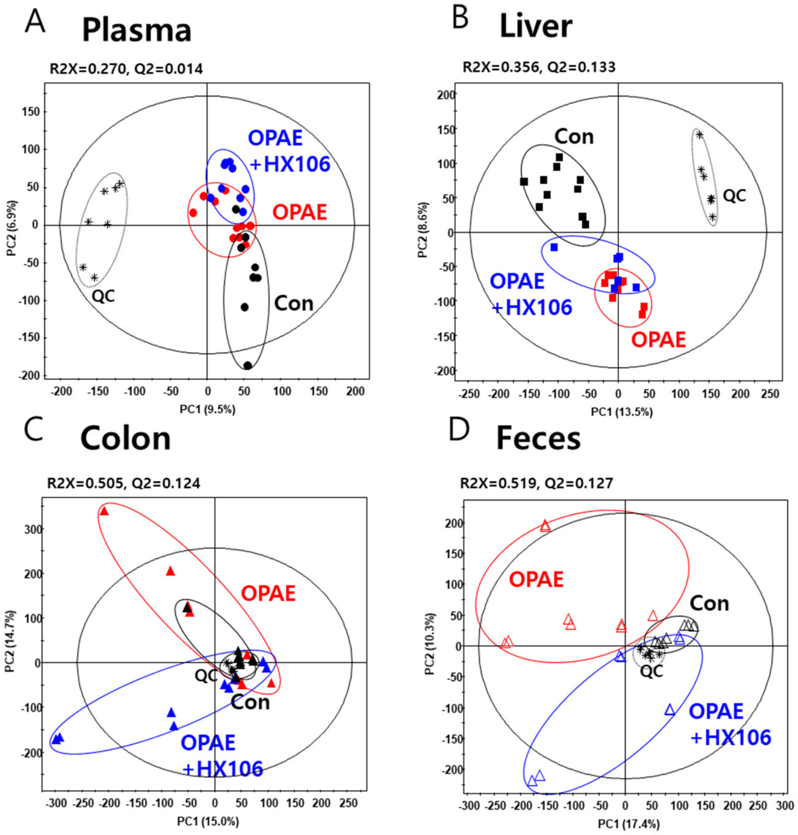

Figure S3. Principal component analysis (PCA) score plot based on metabolic profiling of plasma (**A**), liver (**B**), colon (**C**), and feces (**D**). Con; the control group, OPAE; the offspring affected by PAE.

Table S1. List of discriminated metabolites identified in the plasma. Con; the control group, OPAE; the offspring affected by PAE; OPAE+HX; HX106-treated OPAE group.

No.	Tentative Metabolites[a]	RT (min)	Identified Ion	MS Fragment[b]	TMS	Significance[c]		Fold change[d]		ID[e]
						p-value	FDR	OPAE /Con	OPAE+HX /OPAE	
1	Pyruvic acid	4.80	174	59, 73, 89, 115, 158, 174, 189	1	0.010	0.019	1.181*	1.024	MS/STD
2	Glycolic acid	5.04	147	59, 73, 103, 133, 147, 177, 205	2	0.010	0.019	1.421*	1.421	MS/STD
3	Valine	5.18	72	55, 72, 87, 103, 130, 146, 174	1	0.000	0.000	0.308*	1.343	MS/STD
4	Hydroxylamine	5.43	146	73, 86, 119, 133, 146, 204, 249	3	0.150	0.161	1.164	1.170	STD
5	Oxalic acid	5.62	147	66, 73, 102, 133, 147, 190, 219	2	0.000	0.000	1.641*	1.419*	MS/STD
6	Hydroxyisobutyric acid	5.90	147	73, 88, 101, 117, 130, 133, 147	2	0.000	0.000	1.035	0.584*	STD
7	Isoleucine	6.09	86	57, 75, 86, 103, 130, 146, 188	1	0.000	0.000	0.333*	1.055	MS/STD
8	Succinic acid	7.41	247	55, 73, 129, 133, 147, 172, 247	2	0.001	0.003	0.748*	0.764	MS/STD
9	Threonine	8.13	219	73, 117, 147, 203, 219, 219, 320	3	0.142	0.158	0.810	0.914	MS/STD
10	β-Alanine	8.46	174	73, 100, 133, 147, 174, 248, 290	3	0.137	0.158	1.125	1.026	MS
11	Malic acid	8.99	147	73, 101, 133, 147, 189, 233, 245	3	0.030	0.046	0.947	0.560*	MS/STD
12	Methionine	9.26	176	73, 103, 117, 147, 189, 217, 307	3	0.001	0.004	0.387*	1.198	MS/STD
13	Pyroglutamic acid	9.32	156	59, 73, 100, 147, 156, 230, 258	2	0.000	0.000	2.028*	1.863*	MS/STD
14	Threonic acid	9.62	292	73, 117, 147, 189, 205, 220, 319	4	0.012	0.020	0.744*	1.028	MS
15	α-Ketoglutaric acid	9.71	198	73, 89, 112, 147, 198, 229, 288	1	0.051	0.070	1.030	0.577	MS/STD
16	Glutamic acid	10.04	246	73, 100, 128, 147, 204, 230, 246	3	0.001	0.004	0.440*	1.212	MS/STD
17	Phenylalanine	10.14	218	73, 100, 147, 192, 218, 266, 294	2	0.000	0.000	0.576*	1.082	MS/STD
18	Xylitol	10.87	217	73, 89, 103, 147, 217, 247, 307	5	0.006	0.014	0.786*	0.917	MS/STD
19	Glycerophosphate	11.16	299	73, 147, 211, 256 299, 357, 387	4	0.126	0.153	1.020	1.705	MS
20	Glutamine	11.21	156	73, 83, 128, 147, 156, 203, 245	3	0.204	0.211	1.065	1.001	MS/STD
21	Pinitol	11.71	73	73, 89, 147, 191, 217, 260, 318	5	0.000	0.000	0.430*	2.509*	MS
22	Fructose	11.98	103	73, 89, 103, 147,189, 217, 307	5	0.122	0.153	1.031	1.238	MS/STD
23	Glucose	12.21	205	73, 103, 147, 160, 205, 217, 319	5	0.220	0.220	1.054	0.992	MS/STD
24	Tyrosine	12.38	218	59, 73, 100, 147, 179, 218, 280	3	0.005	0.013	0.719*	0.959	MS/STD
25	Palmitic acid	12.94	117	73, 75, 117, 132, 145, 201, 313	1	0.038	0.056	1.081	0.885	MS/STD
26	*myo*-Inositol	13.41	217	59, 73, 129, 147, 217, 265, 305	6	0.089	0.117	0.775*	1.058	MS/STD
27	Linoleic acid	13.97	150	75, 95, 129, 178, 220, 262, 337	1	0.016	0.026	1.437*	0.969	MS/STD
28	Stearic acid	14.12	117	75, 95, 117, 129, 145, 201, 341	1	0.000	0.001	0.882*	0.927	MS/STD
29	Monopalmitin	16.02	371	73, 103, 129, 147, 203, 239, 371	2	0.009	0.019	0.900*	0.944	MS

[a] Identified metabolites based on variable importance projection (VIP) analysis with cutoff value of 1.0. [b] MS fragmentation is the fragmentation of tentative compound; [c] Significance was analyzed using one-way analysis of variance followed by Tukey's honestly significant difference test. [d] Fold change was calculated by dividing the mean of the peak intensity of each metabolite from each of the two groups. [e] Identification: MS, mass spectrum was consistent with those of NIST and in-house libraries; STD, mass spectrum was consistent with that of standard compound. FDR, false discovery rate; RT, Retention time; TMS, trimethylsilyl; ID, Identification; *FDR-adjusted *p*-value < 0.05.

Table S2. List of discriminated metabolites identified in the liver. Con; the control group, OPAE; the offspring affected by PAE; OPAE+HX; HX106-treated OPAE group.

No.	Tentative Metabolites[a]	RT (min)	Identified Ion	MS Fragment[b]	TMS	Significance[c]		Fold change[d]		ID[e]
						p-value	FDR	OPAE /Con	OPAE+HX /OPAE	
1	Lactic acid	4.89	117	73, 88, 117, 133, 147, 191, 219	2	0.000	0.000	0.919*	1.132*	MS/STD
2	Alanine	5.32	56	59, 73, 100, 116, 133, 147, 190	2	0.058	0.062	1.258	0.598*	MS/STD
3	Hydroxylamine	5.43	146	59, 73, 86, 119, 133, 161, 249	3	0.001	0.002	1.412*	1.051	MS
4	Urea	6.82	189	73, 99, 130, 147, 171, 189, 204	2	0.003	0.004	0.842*	1.069	MS
5	Glycerol	7.07	218	73, 89, 103, 117, 133, 147, 205	3	0.043	0.047	0.938	0.957	MS
6	Adenosine-5'-diphosphate	7.11	225	73, 133, 193, 207, 225, 283, 299	3	0.023	0.027	0.971*	1.048*	MS/STD
7	Succinic acid	7.42	247	73, 116, 129, 147, 172, 218, 247	2	0.005	0.007	0.745*	1.009	MS/STD
8	Fumaric acid	7.71	245	73, 83, 115, 133, 147, 171, 217	2	0.004	0.006	1.268	0.573*	MS/STD
9	Serine	7.88	204	59, 73, 100, 147, 188, 204, 278	3	0.006	0.008	1.305*	0.875	MS/STD
10	Threonine	8.13	219	57, 73, 117, 147, 203, 219, 291	3	0.122	0.122	1.209	0.910	MS/STD
11	β-Alanine	8.46	174	59, 73, 86, 147, 174, 248, 290	3	0.000	0.001	1.382*	0.549*	MS
12	Aminomalonic acid	8.86	71	86, 133, 147, 174, 218, 292, 320	3	0.001	0.002	0.756*	1.515*	MS
13	Malic acid	9.00	233	73, 101, 133, 147, 189, 233, 265	3	0.000	0.000	1.284*	0.531*	MS/STD
14	Aspartic acid	9.26	232	59, 73, 100, 147, 163, 218, 232	3	0.000	0.000	1.831*	0.461*	MS/STD
15	Pyroglutamic acid	9.32	156	59, 73, 133, 147, 156, 230, 258	2	0.000	0.000	1.179*	0.651*	MS/STD
16	Threonic acid	9.62	292	73, 117, 147, 189, 205, 220, 292	4	0.000	0.001	0.716*	0.874*	MS
17	Glutamic acid	10.04	246	73, 128, 147, 230, 246, 348, 363	3	0.000	0.000	1.546*	0.570*	MS/STD
18	Phenylalanine	10.14	218	59, 73, 100, 147, 160, 192, 218	2	0.002	0.004	1.446*	0.817*	MS/STD
19	2-Keto-gluconic acid	10.36	204	73, 103, 117, 133, 147, 204, 217	5	0.012	0.016	0.625*	1.678*	MS
20	Pyrophosphoric acid	10.47	451	73, 133, 193, 207, 299, 263, 451	4	0.000	0.000	2.184*	1.156	MS
21	Xylitol	10.79	217	73, 103, 129, 147, 217, 243, 207	5	0.000	0.000	0.674*	1.319*	MS/STD
22	Glycerophosphate	11.16	299	73, 147, 211, 256, 299, 357, 445	4	0.000	0.001	0.392*	1.608	MS
23	Hypoxanthine	11.52	266	73, 84, 125, 147, 193, 265, 280	2	0.021	0.026	1.233*	0.995	MS
24	Tagatofuranose	11.91	217	73, 103, 147, 191, 217, 257, 437	5	0.000	0.000	0.661*	0.855	MS
25	Fructose	11.98	217	73, 103, 147, 189, 217, 307, 364	5	0.003	0.005	1.051	1.555*	MS/STD
26	Mannose	12.09	160	73, 103, 129, 147, 205, 291, 319	5	0.037	0.042	1.038	1.048	MS/STD
27	Galactose	12.31	160	73, 103, 129, 147, 189, 205, 319	5	0.066	0.068	1.013	1.035	MS/STD
28	Palmitic acid	12.95	117	73, 117, 132, 145, 201, 313, 328	1	0.000	0.000	1.081*	0.866*	MS/STD
29	Oleanitrile	13.24	122	55, 69, 83, 122, 136, 150, 178	-	0.001	0.001	1.711*	1.362	MS
30	*myo*-Inositol	13.41	305	73, 103, 129, 147, 191, 217, 265	6	0.009	0.012	1.144*	1.075	MS/STD
31	Linoleic acid	13.97	150	55, 75, 95, 178, 220, 262, 337	1	0.000	0.000	2.191*	0.526*	MS/STD

[a] Identified metabolites based on variable importance projection (VIP) analysis with cutoff value of 1.0. [b] MS fragmentation is the fragmentation of tentative compound; [c] Significance was analyzed using one-way analysis of variance followed by Tukey's honestly significant difference test. [d] Fold change was calculated by dividing the mean of the peak intensity of each metabolite from each of the two groups. [e] Identification: MS, mass spectrum was consistent with those of NIST and in-house libraries; STD, mass spectrum was consistent with that of standard compound. FDR, false discovery rate; RT, Retention time; TMS, trimethylsilyl; ID, Identification; *FDR-adjusted *p*-value < 0.05.

Table S3. List of discriminated metabolites identified in the colon. Con; the control group, OPAE; the offspring affected by PAE; OPAE+HX; HX106-treated OPAE group.

No.	Tentative Metabolites[a]	RT (min)	Identified Ion	MS Fragment[b]	TMS	Significance[c]		Fold change[d]		ID[e]
						p-value	FDR	OPAE /Con	OPAE+HX /OPAE	
1	Hydroxylamine	5.43	146	59, 73, 86, 100, 119, 133, 146	3	0.120	0.140	1.062	0.696	MS
2	Glycine	5.49	102	59, 73, 102, 131, 147, 176, 204	2	0.248	0.248	1.134	1.370	MS/STD
3	Oxalic acid	5.63	147	73, 117, 133, 147, 175, 190, 219	2	0.000	0.001	1.507*	1.334	MS/STD
4	Leucine	5.87	86	61, 75, 86, 103, 146, 170, 188	1	0.008	0.024	0.406*	1.466	MS/STD
5	Hydroxyisobutyric acid	5.91	147	73, 88, 117, 147, 191, 204, 233	2	0.060	0.083	0.768	0.980	MS
6	Monomethylphosphate	6.14	241	73, 119, 133, 163, 211, 241, 256	2	0.000	0.000	0.936	2.170*	MS
7	Valine	6.49	144	59, 73, 100, 133, 144, 218, 246	2	0.123	0.140	0.628	1.245	MS/STD
8	Urea	6.80	189	73, 99, 130, 147, 171, 189, 204	2	0.198	0.204	2.192	0.687	MS
9	Serine	6.90	116	73, 103, 116, 132, 147, 188, 219	2	0.035	0.062	0.632	1.105	MS/STD
10	Ethanolamine	6.98	174	59, 73, 86, 100, 147, 174, 262	3	0.112	0.138	0.977	0.772	MS/STD
11	Glycerol	7.07	117	73, 103, 117, 133, 147, 177, 205	3	0.010	0.025	1.332	0.699	MS
12	Glyceric acid	7.61	189	73, 133, 147, 189, 205, 292, 307	3	0.064	0.085	1.457	1.113	MS
13	Uracil	7.69	99	73, 99, 113, 126, 147, 241, 256	2	0.033	0.061	1.103	1.094	MS/STD
14	Threonine	8.13	219	73, 101, 117, 147, 219, 291, 320	3	0.028	0.056	0.625*	1.318	MS/STD
15	Thymine	8.26	255	73, 100, 113, 147, 185, 255, 270	2	0.008	0.024	1.289	2.388	MS
16	Aspartic acid	8.43	160	73, 100, 117, 130, 160, 202, 245	2	0.001	0.006	0.555*	1.486	MS/STD
17	Malic acid	9.00	233	73, 101, 147, 189, 233, 265, 335	3	0.016	0.037	0.752*	2.603	MS/STD
18	Threonic acid	9.62	292	73, 117, 147, 189, 220, 292, 319	4	0.001	0.007	0.720	2.244*	MS
19	Phenylalanine	10.14	218	73, 100, 147, 192, 218, 266, 294	2	0.002	0.008	0.587*	1.301	MS/STD
20	Xylose	10.40	217	73, 103, 147, 189, 217, 277, 307	4	0.142	0.152	0.340*	6.018	MS/STD
21	Hypoxanthine	11.50	280	73, 84, 125, 206, 238, 265, 280	2	0.039	0.063	0.835	1.581	MS
22	Fructose	11.98	217	73, 89, 103, 147, 189, 217, 307	5	0.136	0.150	1.403	1.196	MS/STD
23	Lysine	12.23	156	73, 128, 156, 174, 200, 230, 317	4	0.019	0.042	0.278*	1.967	MS/STD
24	Tyrosine	12.36	218	73, 100, 147, 179, 218, 280, 354	2	0.009	0.024	0.506*	1.397	MS/STD
25	Pantothenic acid	12.73	201	73, 103, 157, 201, 218, 247, 291	3	0.056	0.081	1.708*	0.938	MS
26	N-Acetyl-D-glucosamine	13.36	202	73, 87, 103, 129, 147, 202, 319	4	0.000	0.002	2.234*	0.569	MS/STD
27	Linoleic acid	13.98	337	75, 95, 129, 178, 220, 262, 337	1	0.005	0.018	1.025	0.647	MS/STD
28	Oleic acid	14.02	354	73, 75, 117, 199, 264, 339, 354	1	0.002	0.010	1.044	0.712	MS/STD
29	Stearic acid	14.15	341	73, 97, 132, 117, 201, 297, 341	1	0.001	0.007	1.233*	0.638*	MS/STD
30	Glucose 6-phosphate disodium salt hydrate	14.81	387	73, 147, 217, 299, 357, 387, 471	6	0.042	0.064	1.001	1.827	MS/STD
31	*cis*-Docosahexaenoic acid	15.91	224	73 79, 105, 117, 131, 145, 199	1	0.037	0.062	0.776	0.706	MS
32	Docosanoic acid	16.24	132	73, 75, 117, 129, 145, 201, 397	1	0.090	0.116	1.266	0.589	MS

[a] Identified metabolites based on variable importance projection (VIP) analysis with cutoff value of 1.0. [b] MS fragmentation is the fragmentation of tentative compound; [c] Significance was analyzed using one-way analysis of variance followed by Tukey's honestly significant difference test. [d] Fold change was calculated by dividing the mean of the peak intensity of each metabolite from each of the two groups. [e] Identification: MS, mass spectrum was consistent with those of NIST and in-house libraries; STD, mass spectrum was consistent with that of standard compound. FDR, false discovery rate; RT, Retention time; TMS, trimethylsilyl; ID, Identification; *FDR-adjusted *p*-value < 0.05.

Table S4. List of discriminated metabolites identified in the feces. Con; the control group, OPAE; the offspring affected by PAE; OPAE+HX; HX106-treated OPAE group.

No.	Tentative Metabolites	RT (min)	Identified Ion	MS Fragment	TMS	Significance[c]		Fold change[d]		ID[e]
						p-value	FDR	OPAE /Con	OPAE+HX /OPAE	
1	Lactic acid	4.90	219	45, 73, 88, 117, 147, 191, 219	2	0.003	0.010	2.792*	0.535	MS/STD
2	Glycolic acid	5.04	177	59, 73, 103, 133, 147, 177, 205	2	0.124	0.132	1.386*	0.846	MS
3	Glycine	5.49	102	59, 73, 102, 131, 147, 176, 204	2	0.021	0.035	0.492*	1.180	MS/STD
4	Hydroxyisobutyric acid	5.59	131	66, 73, 95, 115, 131, 147, 205	2	0.007	0.019	1.271*	0.856	MS
5	Oxalic acid	5.64	147	66, 73, 102, 133, 147, 190, 219	2	0.000	0.000	0.706*	0.712	MS/STD
6	3-Methyl-2-ketobutyric acid	5.69	202	59, 73, 89, 100, 128, 186, 202	1	0.008	0.020	14.035*	0.434	MS
7	Leucine	5.87	86	61, 75, 86, 103, 130, 146, 188	1	0.023	0.036	0.570*	1.178	MS/STD
8	Isoleucine	6.09	86	75, 86, 103, 130, 146, 170, 188	1	0.032	0.046	0.570*	1.183	MS/STD
9	2-Keto-3-methylpentanoic acid	6.35	89	73, 89, 113, 129, 172, 200, 216	1	0.002	0.010	12.223*	0.427	MS
10	Valine	6.49	144	59, 73, 86, 100, 144, 218, 246	1	0.020	0.034	0.539*	1.134	MS/STD
11	Benzoic acid	6.82	179	51, 77, 105, 121, 135, 179, 194	1	0.053	0.069	1.210*	0.979	MS/STD
12	Serine	6.90	116	73, 103, 116, 132, 147, 188, 219	1	0.139	0.143	0.631*	1.488	MS/STD
13	Glycerol	7.07	218	73, 103, 117, 133, 147, 205, 293	3	0.013	0.027	1.167*	0.797	MS
14	Phosphoric acid	7.09	299	73, 133, 193, 211, 283, 299, 314	3	0.001	0.008	0.723*	1.030	MS
15	Uracil	7.69	256	73, 103, 116, 132, 147, 188, 219	2	0.003	0.010	1.359*	0.810	MS/STD
16	Threonine	8.13	219	73, 101, 117, 147, 219, 291, 320	3	0.042	0.057	0.644*	1.138	MS/STD
17	Thymine	8.27	255	73, 100, 113, 147, 239, 255, 270	2	0.013	0.027	2.183*	0.881	MS
18	Aspartic acid	8.43	160	73, 100, 130, 147, 160, 202, 245	2	0.058	0.073	0.326*	2.893	MS/STD
19	Malic acid	9.00	233	73, 101, 147, 189, 233, 265, 307	3	0.003	0.010	0.437*	1.797	MS/STD
20	Phenylalanine	10.14	218	73, 100, 147, 177, 192, 218, 266	2	0.020	0.034	0.608*	1.114	MS/STD
21	Xylose	10.35	307	73, 89, 103, 147, 189, 217, 233	4	0.100	0.110	0.890	2.108	MS/STD
22	Xylitol	10.78	307	73, 103, 129, 147, 189, 217, 307	5	0.016	0.030	1.387*	1.151	MS/STD
23	Fucose	10.95	117	73, 89, 117, 147, 219, 277, 321	4	0.000	0.001	2.182*	0.533*	MS/STD
24	Pinitol	11.71	260	73, 133, 147, 191, 217, 260, 318	5	0.146	0.146	1.194	3.237	MS
25	Fructose	11.98	217	73, 103, 147, 189, 217, 307, 364	5	0.025	0.038	1.338*	1.036	MS/STD
26	Glucose	12.13	247	73, 103, 117, 147, 189, 205, 319	5	0.079	0.095	1.286*	0.922	MS/STD
27	Tyrosine	12.37	218	73, 100, 147, 179, 218, 280, 354	3	0.092	0.108	0.651*	1.177	MS/STD
28	Palmitic acid	12.95	132	73, 75, 117, 132, 145, 313, 328	1	0.014	0.027	1.054	0.861	MS/STD
29	*cis*-Ferulic acid	13.32	338	73, 147, 219, 249, 293, 323, 338	2	0.000	0.001	0.553*	1.308	MS/STD
30	N-Acetyl-D-glucosamine	13.37	202	73, 87, 147, 173, 202, 243, 274	4	0.000	0.000	3.258*	0.326*	MS/STD
31	*myo*-Inositol	13.43	265	73, 129, 147, 217, 265, 305, 318	6	0.099	0.110	1.100	0.892	MS/STD
32	Oleic acid	14.01	354	73, 75, 117, 145, 199, 264, 339	1	0.008	0.020	1.055*	0.830	MS/STD
33	Stearic acid	14.14	132	55, 73, 75, 117, 129, 201, 341	1	0.000	0.000	1.303*	0.713*	MS/STD
34	Ononitol	15.23	259	73, 103, 129, 147, 204, 217, 259	5	0.003	0.010	0.273*	1.736	MS

[a] Identified metabolites based on variable importance projection (VIP) analysis with cutoff value of 1.0. [b] MS fragmentation is the fragmentation of tentative compound; [c] Significance was analyzed using one-way analysis of variance followed by Tukey's honestly significant difference test. [d] Fold change was calculated by dividing the mean of the peak intensity of each metabolite from each of the two groups. [e] Identification: MS, mass spectrum was consistent with those of NIST and in-house libraries; STD,

mass spectrum was consistent with that of standard compound. FDR, false discovery rate; RT, Retention time; TMS, trimethylsilyl; ID, Identification; *FDR-adjusted *p*-value < 0.05.

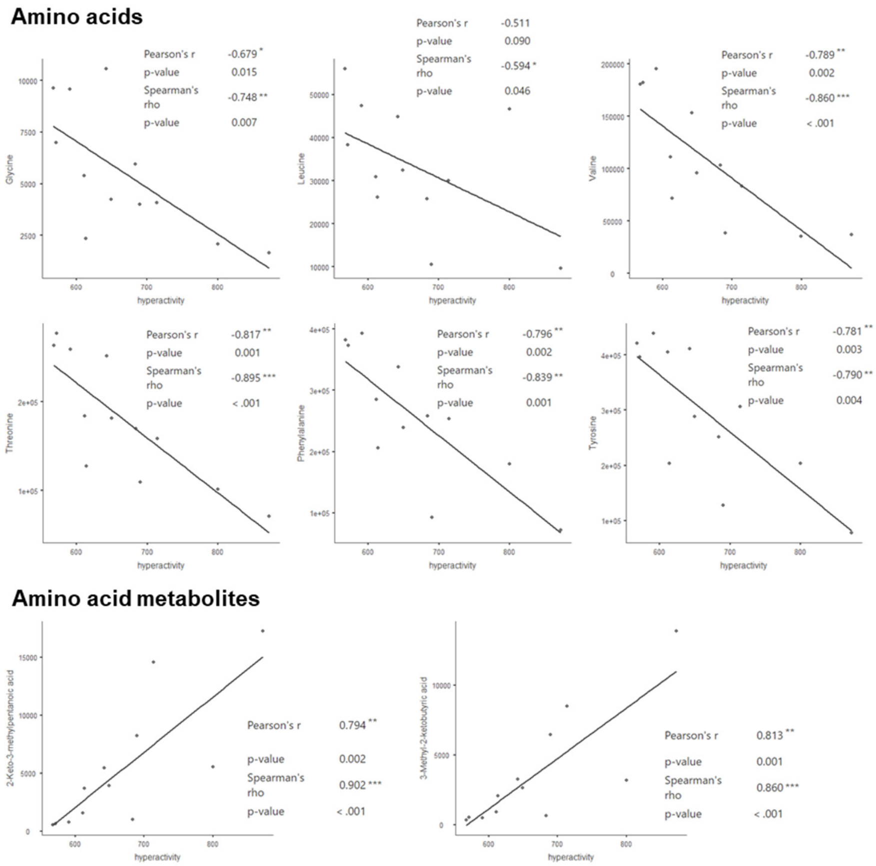

Figure S4. Correlation plot between fecal metabolites with hyperactive behavior. *p<0.05, **p<0.01, and ***p<0.001.

Reference

1. Kim, H.G.; Moon, M.; Choi, J.G.; Park, G.; Kim, A.-J.; Hur, J.; Lee, K.-T.; Oh, M.S. Donepezil inhibits the amyloid-beta oligomer-induced microglial activation in vitro and in vivo. Neurotoxicology, 2014. **40**: p. 23–32.

© 2020 by the authors. Submitted for possible open access publication under the terms and conditions of the Creative Commons Attribution (CC BY) license (http://creativecommons.org/licenses/by/4.0/).
